# The effect of low dose marine protein hydrolysates on short-term recovery after high intensity performance cycling: a double-blinded crossover study

**DOI:** 10.1186/s12970-019-0318-3

**Published:** 2019-10-29

**Authors:** Ingunn Mjøs, Einar Thorsen, Trygve Hausken, Einar Lied, Roy M. Nilsen, Ingeborg Brønstad, Elisabeth Edvardsen, Bente Frisk

**Affiliations:** 1 Department of Health and Functioning, Western Norway University of Applied Sciences, Pb. 7030, 5020 Bergen, Norway; 2 Department of Physiotherapy, Haukeland University Hospital, Bergen, Norway; 3 Dept. of Clinical Science, University of Bergen, Bergen, Norway; 4 Dept. of Occupational Medicine, Haukeland University Hospital, Bergen, Norway; 5 Dept. of Clinical Medicine, University of Bergen, Bergen, Norway; 6 National Centre for Ultrasound in Gastroenterology, Haukeland University Hospital, Bergen, Norway; 7 Firmenich Bjørge Biomarin A/S, Aalesund, Norway; 8 Dept. of Pulmonary Medicine, Oslo University Hospital, Ullevål, Oslo, Norway; 9 Norwegian School of Sport Sciences, Oslo, Norway

**Keywords:** Endurance exercise, Hydrolysed proteins, Marine protein hydrolysate, Recovery

## Abstract

**Background:**

Knowledge of the effect of marine protein hydrolysate (MPH) supplementation to promote recovery after high intensity performance training is scarce. The aim of this study was to examine the effect of MPH supplementation to whey protein (WP) and carbohydrate (CHO): (CHO-WP-MPH), on short-term recovery following high intensity performance, compared to an isoenergetic and isonitrogenous supplement of WP and CHO: (CHO-WP), in male cyclists.

**Methods:**

This was a double-blinded crossover study divided into three phases. Fourteen healthy men participated. In phase I, an incremental bicycle exercise test was performed for establishment of intensities used in phase II and III. In phase II (9–16 days after phase 1), the participants performed first one high intensity performance cycling session, followed by nutrition supplementation (CHO-WP-MPH or CHO-WP) and 4 hours of recovery, before a subsequent high intensity performance cycling session. Phase III (1 week after phase II), was similar to phase II except for the nutrition supplementation, where the participants received the opposite supplementation compared to phase II. Primary outcome was difference in time to exhaustion between the cycling sessions, after nutrition supplementations containing MPH or without MPH. Secondary outcomes were differences in heart rate (HR), respiratory exchange ratio (RER), blood lactate concentration and glucose.

**Results:**

The mean age of the participants was 45.6 years (range 40–58). The maximal oxygen uptake (mean ± SD) measured at baseline was 54.7 ± 4.1 ml∙min^− 1^∙kg^− 1^. There were no significant differences between the two nutrition supplementations measured by time to exhaustion at the cycling sessions (mean_diff_ = 0.85 min, *p* = 0.156, 95% confidence interval (CI), − 0.37, 2.06), HR (mean_diff_ = 0.8 beats pr.min, *p* = 0.331, 95% CI, − 0.9, 2.5), RER (mean_diff_ = − 0.05, *p* = 0.361, 95% CI -0.07 – 0.17), blood lactate concentration (mean_diff_ = − 0.24, *p* = 0.511, 95% CI, − 1.00, 0.53) and glucose (mean_diff_ = 0.23, *p* = 0.094, 95% CI, − 0.05, 0.51).

**Conclusions:**

A protein supplement with MPH showed no effects on short-term recovery in middle-aged healthy male cyclists compared to a protein supplement without MPH.

**Trial registration:**

The study was registered 02.05.2017 at ClinicalTrials.gov (Protein Supplements to Cyclists, NCT03136133, https://clinicaltrials.gov/ct2/show/NCT03136133?cond=marine+peptides&rank=1.

## Background

Appropriate recovery from strenuous exercise is essential both during exercise training and during competitions to maximize physiological adaptations. In cycling, repeated high performance activity is often required after only short time of recovery. Rapid replenishment of energy stores, like muscle and liver glycogen, is therefore necessary, as well as rapid muscle repair and remodelling [1, 2].

Adequate nutrition is vital for optimal recovery, and the importance of protein intake during brief recovery periods is well accepted [3]. However, less is known about the role of protein intake in endurance exercise compared to resistance-based exercise [1].

Protein nutrition is complex, and multiple factors, in addition to the amount of protein ingested, are regarded to be relevant for training adaptations [1, 4–6]. Amino acid composition, digestibility and rate of absorption may differ between proteins strongly affecting their nutritional qualities. During the last decades, whey proteins (WP) have become very popular in sports nutrition, and considered superior to other proteins due to their excellent amino acid profile, high digestibility and the fact that they are rapidly absorbed from the intestine [1]. Recently pre-digested proteins produced by enzymatic hydrolysis turning the protein into peptides [7], has gained interest in sports nutrition due to their more rapidly uptake from the intestine as compared to free amino acids and proteins [7–9]. In addition to rapid supply of amino acids for protein synthesis in tissues, pre-digestion of proteins may produce bioactive peptides specifically affecting secretion of hormones related to recovery, which is not obtained by the undigested protein [10]. Inclusion of hydrolysed proteins in sports nutrition may be beneficial for recovery both by faster regeneration of the glycogen stores, but also by triggering anabolism of protein in muscle tissue [11].

Marine protein hydrolysates (MPH), have gradually gained more attention due to potential health benefits [12], and substantial effects of hydrolysed fish proteins on metabolism have been shown in rats [13, 14]. In a clinical study, comprising 120 overweight male and female subjects, Nobile et al. [15] showed that oral doses of 1.4 and 2.4 g of MPH taken daily for 90 days, significantly affected cholecystokinin (CCK) and glucagon-like peptide-1 (GLP-1). In addition, improved body composition in favour of protein body mass was demonstrated, indicating that MPH show bioactivity in humans when taken orally at doses in the range of 15–20 mg per kg body weight [15].

Two randomized controlled studies with crossover design and great similarities in methods, investigated effects of MPH ingestion during endurance cycling in men [16, 17]. Vegge et al. [17] found no influences of MPH on metabolism. They did however find improved cycling performance in those participants with the lowest aerobic capacity, and thus indicated that MPH provide ergogenic effects in less trained athletes. Interestingly, and contrary to the results found by Vegge et al. [17], Siegler et al. [16] demonstrated metabolic influences of MPH, but could not show effects on performance.

As only a few studies have examined effects of MPH on endurance performance and found discrepant results [16, 17], more knowledge about the potential role of MPH in endurance exercise and high intensity performance is needed. The aforementioned studies were not concerned with recovery, and to our knowledge, no studies have investigated effects of MPH supplementation on recovery after high intensity performance exercise. Furthermore, there has been a lack of evidence on effects of protein nutrition on recovery in middle-aged adults [5]. The main objective of the present study was therefore to examine the effect of MPH supplementation in addition to WP and carbohydrate (CHO) on recovery regarding time to exhaustion after high intensity performance cycling in middle-aged men, compared to an isoenergetic and isonitrogenous supplement of WP and CHO. We hypothesized that low concentrations of MPH, in combination with WP, enhances recovery more than an isonitrogenous amount of WP without MPH.

## Methods

### Study design and procedure

**Fig. 1:**
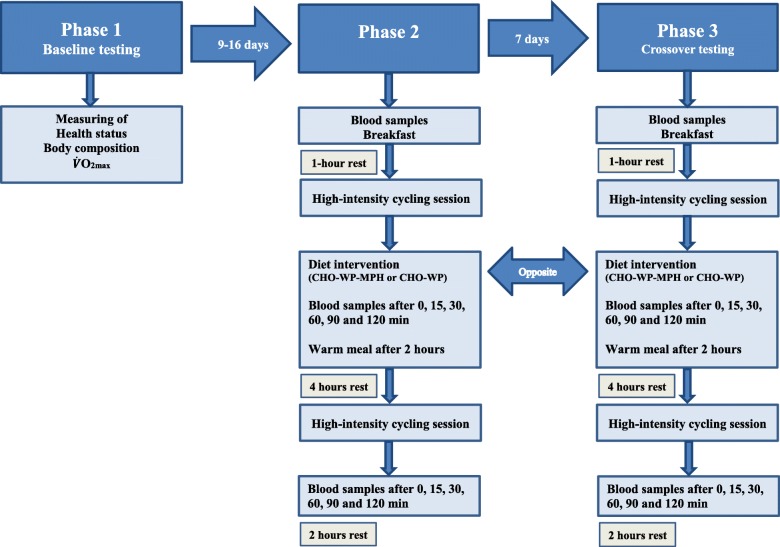
Flowchart of the study. V̇O_2max_: Maximal oxygen uptake

This was a double-blinded crossover study divided into three phases, as shown in Fig. 1, and the study was a part of a larger trial. Assessment of health status, measurement of body composition and testing of maximal aerobic capacity were evaluated in phase I. In phase II, the participants performed a high intensity performance cycling session until exhaustion, before nutrition supplementation and a recovery phase of 4 hours, before a new cycling session equal to the first one was performed. Phase III was similar to phase II except for the nutrition supplementation. There were two alternative diets, composed of WP and CHO, with or without supplementation of MPH (CHO-WP-MPH or CHO-WP). The participants could receive either CHO-WP or CHO-WP-MPH in phase II, and the opposite alternative in phase III. The study was conducted at Western Norway University of Applied Sciences from September to November 2017.

### Participants

Fourteen healthy male volunteers, with cycling as their main exercise activity, were included in the study. They were recruited through advertising in social media, and from local cycle clubs in Bergen and the surrounding municipalities, Norway.

To avoid hormone differences between individuals, no women were recruited. Eligibility criteria were healthy men between 38 and 55 years of age (changed from 40 to 50 years registered in ClinicalTrials.gov), with a body mass index (BMI) from 19 to 29 kg/m^2^, who exercised on average between 8 and 12 h per week the last month prior to inclusion, and at least 70% of the exercise had to be cycling. Exclusion criteria were food allergies, self-reported diabetes mellitus, surgery or trauma with significant blood loss or donation of blood within the last 3 months prior to the study. Musculoskeletal problems that could interfere with their ability to perform the cycling sessions were also cause for exclusion. In addition, participants who had human immunodeficiency virus (HIV), hepatitis B surface antigen (HBsAg), or hepatitis C virus antibody (anti-HCV) and/or had been treated with any investigational drugs, steroids, or medications that effected the intestinal function within 1 month prior to the study or use of antibiotics within 3 months prior to the study were excluded.

The study was conducted according to the declaration of Helsinki and the Western Norway Regional Committee for Medical and Health Research Ethics (REK 2017/56) approved the study. Written informed consent was obtained from all participants prior to inclusion.

### Intervention and procedures

The participants were instructed to abstain from exercise 24 h prior to the testing in phase I, II and III, and they arrived at the laboratory by car or by public transportation. They were recommended to maintain approximately the same training frequency, volume and intensity between phase II and III, as in the last week before phase II. In addition, they were told not to drink more than five cups of coffee per day during the study period and to refrain from alcohol 48 h prior to each visit.

### Phase I

#### Health status

Health status was assessed based upon a self-reported questionnaire and a further evaluation when necessary as judged by the physician.

#### Body composition

Height and weight were recorded, including measurement of body composition by use of InBody 720 (InBody Co., Ltd., Cerritos, California, USA). Measurements of body composition included total body weight and height, BMI, fat mass, fat free mass and muscle mass. The BMI was calculated as the body mass divided by the square of height. Measurements were conducted without shoes and socks, and the participants were wearing cycling clothes.

#### Incremental step exercise test

An incremental step exercise test was conducted on a bicycle ergometer to establish the relationship between workload (Watt/W) and oxygen uptake (V̇O_2_), and to measure maximal oxygen uptake (V̇O_2max_) (Jaeger Oxycon Pro GmbH, Würzburg, Germany).

The test started with a warm-up phase at 100 W for 8 min. The workload was then increased by 25 W every 4th min until the blood lactate threshold (LT) was reached. The LT was defined as 1.5 mmol/L above the lowest blood lactate level measured according to methods described by Borch et al. [18]. The cycling was performed with a pedal frequency of 90 revolutions per minute (rpm). Tidal volume (V_T_), breathing frequency (B_f_), V̇O_2_, carbon dioxide output (V̇CO_2_) and respiratory exchange ratio (RER) were measured during a period of 60–90 s on each workload. At the end of each workload, heart rate (HR) (Polar Electro OY, Kempele, Finland or Garmin Edge 1000, Garmin Ltd., Schaffhausen, Switzerland) and perceived exertion by use of the Borg RPE scale 6–20 («rating of perceived exertion», RPE) [19] were registered. After each step a measure of capillary blood lactate and glucose were taken from the fingertip and immediately analysed (Biosen C-Line, EKF Diagnostics Holdings plc, Cardiff, United Kingdom).

After reaching a blood lactate level of 1.5 mmol/L above the lowest measure, the test for V̇O_2max_ was performed immediately by increasing the workload with 25 W every 30 s until exhaustion. During this maximal exercise test, V_T_, B_f_, V̇O_2_, V̇CO_2_, RER and HR were measured continuously until exhaustion, and at exhaustion the Borg RPE was registered immediately, as well as measurements of blood lactate and glucose.

The participants were cycling either on a Lode Excalibur Sport ergometer (Lode B.V., Groningen, The Netherlands), or on a Velotron bicycle ergometer (RacerMate Inc., Seattle, Washington). Each participant performed every cycling session on the same bike throughout the study. In addition, all individual adjustments for seating position, like the height and angle of the saddle and the handlebar, were identical every time for the same participant.

Gas exchange and ventilatory variables during all cycling sessions were measured using a mixing chamber. The minute ventilation was corrected to the body temperature pressure saturated condition, and V̇O_2_ and V̇CO_2_ to the standard temperature pressure dry condition.

### Phase II

In phase II, 9–16 days after phase I, the participants performed two high intensity performance cycling sessions with nutrition supplementation and 4 hours of recovery between the sessions.

Phase II involved the following procedures: The participants had a standardized light breakfast meal 1 hour prior to the first high intensity cycling session. Immediately after the cycling session, the participants ingested the nutrition supplementation. After 4 hours of recovery, the cycling session was repeated.

Following both cycling sessions, venous blood samples were taken after 0, 15, 30, 60, 90 and 120 min. At similar time intervals, the participants filled out questionnaires regarding hunger, satiety, abdominal pain, nausea, diarrhoea and desire to eat. Urine was collected during the whole day. Results from these measurements are beyond the scope of this article and will not be presented here.

Two hours into the four-hour recovery period, the participants were served a standardized warm meal. They were allowed to drink a total of 2.5–3 l of water during the day.

#### High intensity performance cycling sessions

The cycling sessions were initiated with a 20 min moderate intensity at 60% of V̇O_2max_. The exercise load was then increased directly to 90% of V̇O_2max_ for 5 min. Finally, the participants were cycling on a workload corresponding to 95% of V̇O_2max_ until exhaustion. Linear regression analyses were used to determine the relationship between the workload (W) and V̇O_2_ measured in phase I, and the W at the given intensities relatively to V̇O_2max_ were further determined based on individual V̇O_2max_ values. The participants were instructed to keep a pedalling frequency of 90 rpm, and exhaustion was achieved when the frequency fell below 80 rpm. The time to exhaustion performed at 95% of V̇O_2max_ was registered. V̇O_2_, V̇CO_2_ and RER were measured between 9 and 10 min at 60% of V̇O_2max_, and between 3 and 4 min at 90% of V̇O_2max_. HR and Borg RPE were registered every 5 min throughout the cycling sessions, and at exhaustion. Blood lactate concentration and glucose were measured before and immediately after each high intensity cycling session.

The participants were blinded for time to exhaustion when cycling at 95% of V̇O_2max_.The cycling sessions were supervised by experienced technicians. The participants were informed about rpm during the sessions when needed, but to obtain high test-retest reliability, there was no cheering or encouragement during the cycling sessions.

#### Nutrition supplementation

Participants reported to the laboratory in the fasted state. They received a standardised breakfast meal comprising a baguette of semi-coarse bread (93 g) with ham (25 g), white cheese (33 g), no butter, coffee (200 mL) and a glass (200 mL) of orange juice, in total 450 kcal and 22 g protein (19.5% (protein energy/total energy) followed by 1 hour rest before the first cycling session. Immediately after the bout, the participants ingested the test or placebo drink, followed by blood sampling (T = 0), and then sampling at intervals for 120 min whilst resting. Then they received a ready-to-use hot meal (Beef Stroganoff with rice, produced by Fjordland, Norway), containing 450 kcal distributed between 57% CHO, 25% protein and 18% fat, whilst resting for another 2 hours before entering the second cycling bout. The participants were allowed to drink a total of 2.5–3 l of water throughout the intervention day.

The nutrition supplementations CHO-WP (placebo) and CHO-WP-MPH (test) were given in the form of a powder dissolved in water. The powders contained 4.2 kcal/gram distributed, in terms of total energy, between 12% from protein, 66% from CHO, and 22% from fat. WP (WPC80/TINE, Norway) was used as the basic source of protein, while the sources of CHO and fat were, respectively, maltodextrin (DE 20) from corn, and vegetable medium chain triglyceride (MCT) powder (BERGAMAST), i.e. MCT coated with maltodextrin at the ratio 70:30, respectively. The powders were slightly acidified with citric acid and flavoured with a strawberry flavouring agent (Firmenich SA, Switzerland) to level out any differences in terms of taste or smell. The serving size of the powders was standardized to 80 kg body weight providing 295 kcal in 70 g of powder giving 3.68 kcal/kg bodyweight, and 20 mg MPH in the test powder equal to a serving size of 1.600 mg in terms of protein (Nx6.25). The placebo powder was made by replacing MPH with equal amounts of WPC80 in terms of protein (Nx6.25) making the powders both isonitrogenous and isoenergetic. By adjusting the amount of powder to their body weight each participant was given equal amounts of MPH-protein or placebo-protein (WPC80) as well as total protein, carbohydrate, fat and energy in terms of body weight. The difference in the amino acid profiles between MPH and WP was considered insignificant. The beverages were made by dissolving powder in cold water at a ratio 1:2 30 min prior to use to form creamy drinks.

The MPH was provided by Firmenich Bjorge Biomarin AS, Ellingsoy/Norway, and was industrially produced by enzymatic hydrolysis of fresh frozen meat from Atlantic cod (*Gadus morhua*) using the food approved enzyme preparation Protamex® (Novozymes, Copenhagen). The hydrolysate was spray-dried into a powder containing 89% crude protein and < 0.5% fat. The molecular weight (MW) profile of the MPH was analysed by Firmenich-Geneve/Switzerland using size exclusion chromatography (Supradex Peptide 10/300 GL (GE Healthcare, Uppsala-Sweeden)) and UV detection (SEC/UV), and free amino acids by HPLC and Waters Pico-Tag method using UV detection. The analyses showed that about 90% of the peptides had MW less than 2.000 Da (i.e. 18 amino acids or less), about 75% with MW less than 1000 Da (i.e. 10 amino acids or less), and 55% with MW less than 500 Da (i.e. 5 amino acids or less). Twenty-five to 30% of the peptides had MW less than 200 Da representing small dipeptides and free amino acids, the latter accounting for 4.5% of the hydrolysate.

#### Procedures and blinding

The nutrition supplementations were provided, randomly numbered, from the manufacturer (Firmenich Bjørge Biomarin AS, Aalesund/Norway). An experienced biochemist was responsible for composition and blinding of the diets. In phase II, the participants chose one of two alternative drinks, from identically looking bottles, hereby determining the sequence of the diets. In phase II, five participants chose drinks containing MPH, and nine in phase III. The technicians and the participants were all blinded for the contents throughout the study, and the researchers were blinded during the statistical analyses.

### Phase III

The participants returned for crossover testing after a washout period of seven days to repeat the procedures described in phase II. The time of the day was the same for each participant as they met at the same time in the morning in phase II and III in order to avoid circadian variance. The only difference from the protocol was the administration of the alternative beverage.

### Outcome measures

Primary outcome in this subanalysis was differences in performance between cycling sessions after diets with MPH compared to diets without MPH, measured by time to exhaustion at 95% of V̇O_2max_. Secondary outcomes were differences in HR, RER, glucose and blood lactate concentration after diets with MPH compared to diets without MPH.

### Statistics

Because less is known about MPH and possible ergogenic effects, compared to indications from previous studies regarding influences of MPH on glucose [20, 21], power estimation in the main trial was calculated based on blood sugar profile. With an estimated change in mean blood sugar profile (area under the curve) of 20%, power of 80%, type 1 error of 0.05 and a standard deviation of 10% the power calculations estimated that 14 participants had to be included in the study.

Descriptive statistics were used to characterize the participants (mean, standard deviation (SD) median and percent). Paired samples *t* tests were used for comparison between cycling sessions and between the sequences of the nutrition supplementations, CHO-WP versus CHO-WP-MPH (mean, SD and 95% confidence interval (CI)). The outcome variables were differences in cycling time at 95% of V̇O_2max_, RER measured at 90% of V̇O_2max_, and HR, glucose and blood lactate measured at the end of the cycling sessions in the morning versus in the afternoon were compared.

We did not ensure equal distribution of CHO-WP-MPH and CHO-WP in phase II and III. However, we found no period or sequence effects on the various outcomes.

The significance level was set at 0.05. The statistical analyses were carried out using IBM SPSS Statistics 24 for windows (SPSS Inc., Chicago, Illinois, USA) and R version 3.4.1 (The R Foundation for Statistical Computing, www.r-project.org).

## Results

### Participants

Fourteen men were included and all participants completed all phases as planned. Characteristics of the participants at baseline are presented in Table 1. Summarized, mean age was 45.6 ± 5.3 years (range 40–58), and BMI was 24.5 ± 2.2 kg/m^2^. The mean exercise capacity, measured as V̇O_2max,_ was 54.7 ± 4.1 ml∙min^− 1^∙kg^− 1^, the mean workload at maximal exertion was 422 ± 32 W and the median Borg RPE was 19 at the end of the test.

**Table 1:** Baseline characteristics of the participants and physiological responses to the incremental exercise test on treadmill

Characteristics (*N* = 14)	Mean
Age (years)	45.6 ± 5.3
Height (cm)	181 ± 4
Weight (kg)	80.1 ± 6.4
BMI (kg/m^2^)	24.5 ± 2.2
Muscle mass (kg)	37.7 ± 2.3
Fat mass (%)	16.6 ± 4.4
V̇O_2max_ (ml∙min^−1^∙kg^− 1^)	54.7 ± 4.1
Workload_max_ (Watt)	422 ± 32
RER_max_	1.20 ± 0.10
V̇_Emax_ (L/min)	167 ± 16
Lactate_max_ (mmol/L)	11.2 ± 1.4
HR_max_ (bpm)	185 ± 8
Glucose_max_ (mmol/L)	4.8 ± 1.1
Borg RPE_max_ (median)	19

Data are presented as mean ± standard deviation (SD) unless otherwise stated. BMI: body mass index; V̇O_2max_: maximal oxygen uptake; RER: respiratory exchange ratio; V̇_E_: ventilation; HR: heart rate; RPE: rating of perceived exertion

### High intensity performance cycling sessions

The average workloads (W) at the high intensity performance cycling sessions were 174.7 ± 22.1 W, 301.3 ± 31.3 W and 322.5 ± 32.9 W at 60, 90 and 95% of V̇O_2max_ respectively. The median Borg RPE registered at exhaustion was 19 in both morning and afternoon cycling sessions in phase II, and 19 and 20 in morning and afternoon cycling sessions respectively in phase III.

Results from the high intensity performance cycling sessions (phase II and III) in the morning and in the afternoon are presented in Table 2. When CHO-WP-MPH was consumed, the cycling time at 95% of V̇O_2max_ was 6.2 ± 4.6 min (total cycling time 31.2 ± 4.6 min) in the morning sessions and 4.8 ± 3.1 min (total cycling time 29.8 ± 3.1 min) in the afternoon sessions, a reduction in time of 1.4 ± 2.0 min (*p* = 0.026). When CHO-WP was consumed, the cycling time at 95% of V̇O_2max_ was 6.0 ± 4.5 min (total cycling time 31.0 ± 4.5 min) and 5.5 ± 4.6 min (total cycling time 30.5 ± 4.6 min) in the morning and in the afternoon sessions respectively, giving a reduction time of 0.5 ± 1.2 min (*p* = 0.121). Lactate (*p* < 0.001) and glucose (*p* = 0.015) were lower in the morning compared to the afternoon exercise sessions in both conditions (Table 2). Neither HR nor RER changed significantly after recovery in either condition.

**Table 2:** Morning and afternoon cycling sessions when CHO-WP-MPH and CHO-WP were consumed

	CHO-WP-MPH			CHO-WP		
	*N* = 14			*N* = 14		
	Morning	Afternoon		Morning	Afternoon	
	Mean ± SD	Mean ± SD	*p-*value	Mean ± SD	Mean ± SD	*p-*value
Time at 95% of V̇O_2max_ (min)^1,2^	6.2 ± 4.6	4.8 ± 3.1	0.026	6.0 ± 4.5	5.5 ± 4.6	0.121
HR (bpm)^2^	181 ± 8	180 ± 8	0.166	182 ± 7	180 ± 7	0.052
RER^3^	1.06 ± 0.04	1.06 ± 0.04	0.486	1.05 ± 0.05	1.11 ± 0.22	0.331
Lactate (mmol/L)^2^	13.1 ± 2.1	11.3 ± 2.3	< 0.001	13.2 ± 2.0	11.1 ± 2.2	< 0.001
Glucose (mmol/L)^2^	5.4 ± 1.4	4.6 ± 1.1	0.001	5.2 ± 1.5	4.6 ± 1.0	0.015

Data are presented as mean values± standard deviations (SD). The *P*-value represents the significance level of the difference between the morning and the afternoon cycling sessions for CHO-WP-MPH and CHO-WP, respectively. CHO, carbohydrate; WP, whey protein; MPH, marine protein hydrolysate; V̇O_2max_, maximal oxygen uptake; HR, Heart rate; RER, respiratory exchange ratio. ^1^Performance at cycling sessions measured as time to exhaustion (min) when cycling at a workload corresponding to 95% of V̇O_2max_. ^2^Measured at the end of the cycling sessions. ^3^RER measured at 90% of V̇O_2max_

### CHO-WP-MPH compared to CHO-WP

When comparing the differences between the high intensity performance cycling sessions in the morning and afternoon in the CHO-WP-MPH condition to the differences between morning and afternoon sessions in the CHO-WP condition, there were no significant differences between conditions regarding time to exhaustion at 95% of V̇O_2max_, RER, lactate, glucose or HR (Table 3).

**Table 3:** Differences between morning minus afternoon cycling sessions for CHO-WP-MPH and CHO-WP and comparison of the diets

	^a^CHO-WP-MPH	^a^CHO-WP	Diff. CHO-WP-MPH versus		
	*N* = 14	*N* = 14	CHO-WP		
	Mean _diff_ ± SD	Mean _diff_ ± SD	Mean _diff_	95% CI	*p-*value
^b^Time_diff_ at 95% of V̇O_2max_ (min)	1.37 ± 2.03	0.52 ± 1.17	0.85	−0.37, 2.06	0.156
HR (bpm)	−0.9 ± 2.4	−1.7 ± 3.0	0.8	−0.9, 2.5	0.331
RER	−0.01 ± 0.03	−0.06 ± 0.21	− 0.05	−0.07, 0.17	0.361
Lactate (mmol/L)	1.88 ± 0.83	2.12 ± 1.02	−0.24	−1.00, 0.53	0.511
Glucose (mmol/L)	0.78 ± 0.65	0.55 ± 0.73	0.23	−0.05, 0.51	0.094

Data are presented as mean values, standard deviations (SD), 95% confidence interval (CI), and *P*-value. Diff. CHO-WP-MPH versus CHO-WP: differences between morning and afternoon cycling sessions with ingestion of CHO-WP-MPH versus CHO-WP. ^a^ Five participants ingested CHO-WP-MPH and nine CHO-WP in the first intervention (phase II) and in the second intervention (phase III) nine participants ingested CHO-WP-MPH and five CHO-WP. ^b^ Time_diff_ at 95% of V̇O_2max_,: differences between cycling time in the morning and in the afternoon at 95% of V̇O_2max_. CHO, carbohydrate; WP, whey protein; MPH, marine protein hydrolysate; _diff_, difference; HR, Heart rate; bpm, beats pr. min; RER, respiratory exchange ratio

**Fig. 2:**
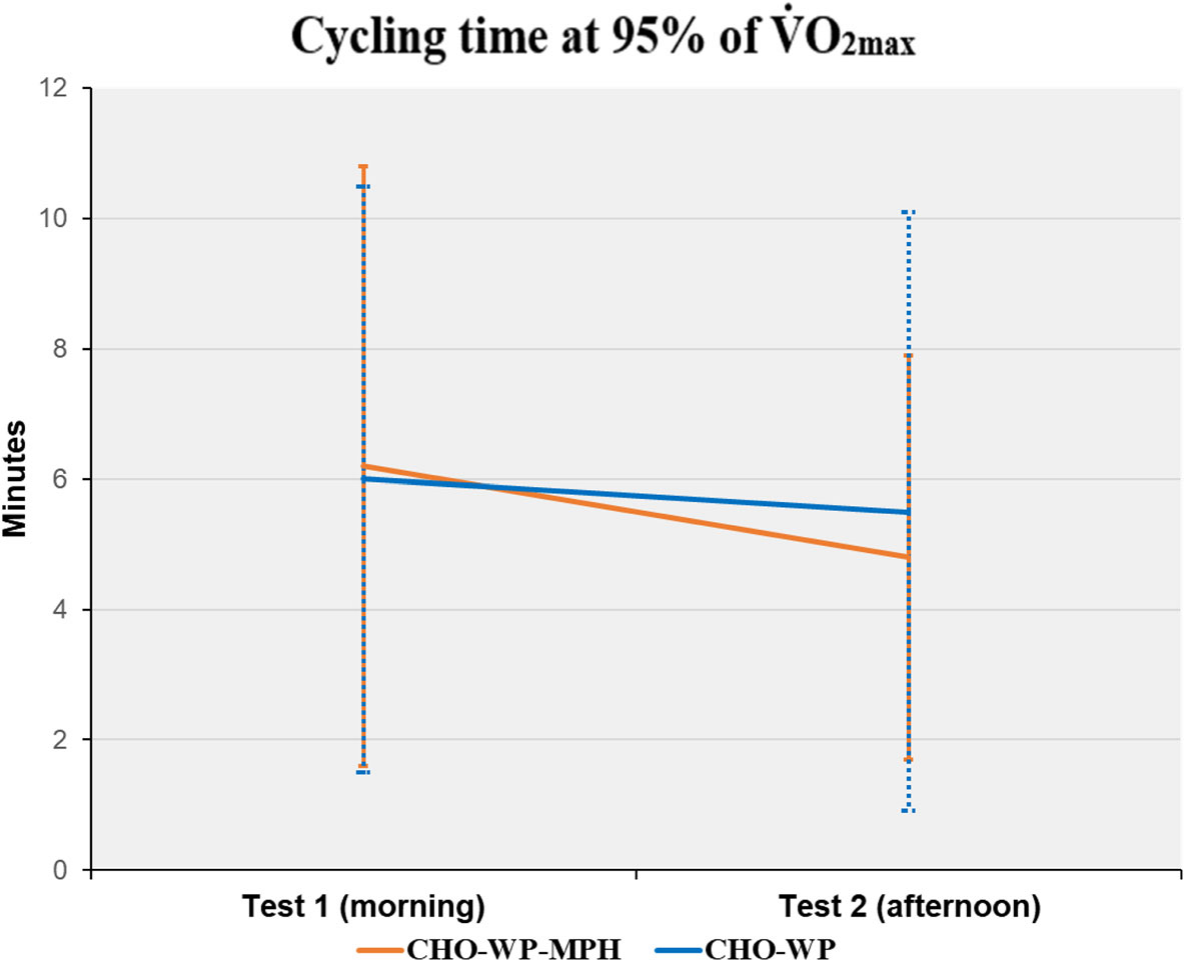
Mean difference in time between morning and afternoon cycling sessions. V̇O_2max_: maximal oxygen uptake; CHO: carbohydrate; WP: whey protein; MPH: marine protein hydrolysate

Cycling performance was, with the exception of two participants, better in the morning compared to the afternoon. Time to exhaustion at 95% of V̇O_2max_ was less reduced in the afternoon when CHO-WP had been consumed (11 ± 26%), compared to CHO-WP-MPH (20 ± 18%). However, the difference of 9% (95% CI,-4.65, 22.29) between conditions was not significant (*p* = 0.181). The difference in performance between morning and afternoon cycling sessions are reported in minutes in Fig. 2 and Table 3.

## Discussion

We examined the effect of MPH supplementation on recovery after high intensity performance cycling. The main finding was that supplementation with MPH in addition to WP and CHO could not improve recovery after high intensity performance cycling in middle-aged men compared to supplementation with CHO-WP.

The cycling time at 95% of V̇O_2max_ was less reduced in the afternoon sessions when CHO-WP had been consumed compared to CHO-WP-MPH. This could possibly indicate that the beverage without MPH was most effective. However, the difference was not consistently observed across participants, and the statistical analysis did not provide evidence that MPH influenced recovery in either direction (*p* = 0.181).

To our knowledge, this is the first study to examine if MPH from Atlantic cod has effect on recovery after high intensity performance cycling. A major strength of the present study is the comparison of isoenergetic and isonitrogenous beverages. WP has a high rate of digestibility and stimulates muscle protein synthesis after exercise more than other protein sources, and the superior effects of WP on recovery compared to other protein sources are well established [1, 5]. Based on the results from Chevrier et al. [20], we hypothesized that MPH in low concentrations could act synergistically with WP, thereby enhancing and accelerating recovery processes beyond what could be expected of CHO and WP alone. Physiological effects of small concentrations of fish protein hydrolysate on metabolism have been observed [20], and the current dose of MPH was hypothesized to be sufficient to achieve bioactivity [15]. However, in this study, additional effects of MPH were not found. Since the beverages in both conditions had a high content of WP, in addition to CHO, and only a small part (3.2%) of the WP was replaced by MPH in the CHO-WP-MPH beverage, it is possible that the recovery processes influenced by nutrition were already optimal without MPH, or that the current dose of MPH was not sufficient.

In addition, we aimed to replicate common post-exercise circumstances in the recovery period, and the cyclists therefore consumed a warm meal after 2 hours of recovery. This already reasonable nutrition, in addition to the optimal content of CHO and WP in both beverages, is regarded a great strength of this study, as it represents habitual post-exercise strategies. However, these nutritional strategies could represent limitations as well, because effects of MPH in small concentrations could be difficult to reveal under otherwise optimal circumstances. Future studies investigating MPH should probably consider different protein source compositions, possibly including larger doses of MPH as well.

With attention to the experimental design of the present study, both the high intensity performance cycling protocols with relatively short durations, and the recovery period of only 4 hours, need to be discussed. As for the former, it could be questioned whether the cycling sessions were demanding enough in order to expect benefits from the nutrition supplementations. Cycling at 95% of V̇O_2max_ led to exhaustion, and blood lactate levels, HR and Borg RPE confirmed that the high intensity performance cycling sessions in the morning were quite demanding for the participants.

In several previous studies where effects of protein supplements on recovery after cycling have been examined, the cycling protocols had longer duration compared to our study, aiming not only to exhaust the cyclists, but to empty glycogen stores as well [22–24]. With a total duration of 31.1 ± 4.4 min at morning sessions in the current study, including 20 min performed at an intensity corresponding to 60% of V̇O_2max_, we could not expect glycogen depletion [25], nor did we aim to deplete endogenous fuel stores. In addition, any metabolic effects of fish protein hydrolysates [13, 14] could be difficult to confirm with a cycling protocol of relatively short duration, which does not depend much on fatty acid metabolism. We aimed to examine ergogenic effects after short time of recovery and wanted a corresponding cycling protocol of short duration. It is reasonable to believe that the relatively short duration and high intensity ensured that fatigue occurred when physiological limits were reached, not influenced by psychological processes and motivation. Therefore, we assume that the reliability of measurements from the exercise sessions was high. In addition, the procedures related to the high intensity performance cycling sessions ensured blinding of time at 95% of V̇O_2max_, as well as strict regulation of verbal instructions. This increased the methodological strength and reduced the possibility that motivation could influence outcomes in this study.

As previously mentioned, the recovery period of only 4 hours in the current study is relatively short. Several studies have used longer periods of recovery when investigating effects of nutritional supplements on recovery, for example 12–15 h [26], 18 h [27], and 24–72 h [28]. However, shorter recovery periods have been described as well, and effects of protein ingestion in combination with CHO on recovery have been demonstrated after three [29] and 4 hours of recovery [24]. The relatively short recovery time can represent a limitation in our study. Still, it could just as well be an advantage, as protein supplements could be of greater benefit with regard to protein synthesis and glycogen repletion when recovery time is insufficient [3].

We did not perform block randomization, which may be a limitation. Five participants consumed CHO-WP-MPH in phase II and nine in phase III. However, when controlling for both sequence and period effects, the statistical analyses demonstrated no significant differences if CHO-WP-MPH was taken in phase II or in phase III.

Our power estimation for the main trial was based on the effects of MPH on blood sugar. Therefore, when investigating ergogenic effects in this subanalysis, we cannot be completely sure that we had enough participants. However, compared to several other studies [16, 17, 22, 27, 30], 14 participants seems to be more than what is commonly described. In addition, the crossover design ensures that relatively few participants are required and the participants serve as their own control [31].

With regard to interpretation of results, the level of aerobic capacity in this study must be taken into account. A Norwegian national cohort study [32] reported reference values for V̇O_2max_ to be 42.7 ± 9.3 and 36.8 ± 6.6 for men aged 40–49 and 50–59, respectively. Compared to this, the participants in our study, with a mean V̇O_2max_ of 54.7 ± 4.1 ml∙min^− 1^∙kg^− 1^, had a high aerobic capacity. This corresponds well to our inclusion criteria, requiring a large amount of weekly exercise. However, the level of fitness in relation to possible effects of MPH is a matter of further interest. Vegge et al. [17], with their study investigating ingestion of MPH during endurance cycling, found indications that effects of MPH could be related to aerobic capacity. Interestingly, they found that the participants with a lower aerobic capacity performed best after ingestion of MPH [17]. A suggestion for future studies could therefore be to include less trained participants when exploring effects of MPH.

## Conclusions

In conclusion, the current study did not reveal effects of low dose MPH supplementation in addition to WP and CHO, compared to an isoenergetic and isonitrogenous supplement of WP and CHO on recovery after high intensity performance cycling in well-trained middle-aged men.

## Data Availability

The datasets used and/or analysed during the current study are available from the corresponding author on reasonable request.

## Abbreviations

B_f_: Breathing frequency
BMI: Body mass index
CHO: Carbohydrate
CO_2_: Carbon dioxide
Da: Dalton
HR: Heart rate
MCT: Medium chain triglyceride
MPH: Marine protein hydrolysate
MW: Molecular weight
O_2_: Oxygen
RER: Respiratory exchange ratio
RPE: Ratings of perceived exertion
rpm: Pedal frequency (“revolutions per minute”)
V̇CO_2_: Carbon dioxide output
V̇_E_: Ventilation
V̇O_2_: Oxygen uptake
V̇O_2max_: Maximal oxygen uptake
V_T_: Tidal volume
W: Workload/Watt
WP: Whey protein
