# Correction to: The effect of low dose marine protein hydrolysates on short-term recovery after high intensity performance cycling: a double-blinded crossover study

**DOI:** 10.1186/s12970-019-0324-5

**Published:** 2020-01-03

**Authors:** Ingunn Mjøs, Einar Thorsen, Trygve Hausken, Einar Lied, Roy M. Nilsen, Ingeborg Brønstad, Elisabeth Edvardsen, Bente Frisk

**Affiliations:** 1 Department of Health and Functioning, Western Norway University of Applied Sciences, Pb. 7030, 5020 Bergen, Norway; 2 Department of Physiotherapy, Haukeland University Hospital, Bergen, Norway; 3 Department of Clinical Science, University of Bergen, Bergen, Norway; 4 Department of Occupational Medicine, Haukeland University Hospital, Bergen, Norway; 5 Department of Clinical Medicine, University of Bergen, Bergen, Norway; 6 National Centre for Ultrasound in Gastroenterology, Haukeland University Hospital, Bergen, Norway; 7 Firmenich Bjørge Biomarin A/S, Aalesund, Norway; 8 Department of Pulmonary Medicine, Oslo University Hospital, Ullevål, Oslo, Norway; 9 Norwegian School of Sport Sciences, Oslo, Norway

**Correction to: J Int Soc Sports Nutr (2019) 16:48**

**https://doi.org/10.1186/s12970-019-0318-3**

The original article [1] contains errors in Tables 1 and 3: Table 1 erroneously mentions use of a treadmill which should instead state ‘bicycle’, and Table 3 has a minor typesetting mistake.

**Table 1:** Baseline characteristics of the participants and physiological responses to the incremental exercise test on bicycle

Characteristics (*N* = 14)	Mean
Age (years)	45.6 ± 5.3
Height (cm)	181 ± 4
Weight (kg)	80.1 ± 6.4
BMI (kg/m^2^)	24.5 ± 2.2
Muscle mass (kg)	37.7 ± 2.3
Fat mass (%)	16.6 ± 4.4
V̇O_2max_ (ml∙min^−1^∙kg^− 1^)	54.7 ± 4.1
Workload_max_ (Watt)	422 ± 32
RER_max_	1.20 ± 0.10
V̇_Emax_ (L/min)	167 ± 16
Lactate_max_ (mmol/L)	11.2 ± 1.4
HR_max_ (bpm)	185 ± 8
Glucose_max_ (mmol/L)	4.8 ± 1.1
Borg RPE_max_ (median)	19

Data are presented as mean ± standard deviation (SD) unless otherwise stated. BMI: body mass index; V̇O_2max_: maximal oxygen uptake; RER: respiratory exchange ratio; V̇_E_: ventilation; HR: heart rate; RPE: rating of perceived exertion

**Table 3:** Differences between morning minus afternoon cycling sessions for CHO-WP-MPH and CHO-WP and comparison of the diets

	^a^CHO-WP-MPH	^a^CHO-WP	Diff. CHO-WP-MPH versus CHO-WP		
	*N* = 14	*N* = 14			
	Mean _diff_ ± SD	Mean _diff_ ± SD	Mean _diff_	95% CI	*p-*value
^b^Time_diff_ at 95% of V̇O_2max_ (min)	1.37 ± 2.03	0.52 ± 1.17	0.85	−0.37, 2.06	0.156
HR (bpm)	−0.9 ± 2.4	−1.7 ± 3.0	0.8	−0.9, 2.5	0.331
RER	−0.01 ± 0.03	−0.06 ± 0.21	− 0.05	− 0.07, 0.17	0.361
Lactate (mmol/L)	1.88 ± 0.83	2.12 ± 1.02	−0.24	−1.00, 0.53	0.511
Glucose (mmol/L)	0.78 ± 0.65	0.55 ± 0.73	0.23	−0.05, 0.51	0.094

Data are presented as mean values, standard deviations (SD), 95% confidence interval (CI), and *P*-value. Diff. CHO-WP-MPH versus CHO-WP: differences between morning and afternoon cycling sessions with ingestion of CHO-WP-MPH versus CHO-WP. ^a^ Five participants ingested CHO-WP-MPH and nine CHO-WP in the first intervention (phase II) and in the second intervention (phase III) nine participants ingested CHO-WP-MPH and five CHO-WP. ^b^ Time_diff_ at 95% of V̇O_2max_,: differences between cycling time in the morning and in the afternoon at 95% of V̇O_2max_. CHO, carbohydrate; WP, whey protein; MPH, marine protein hydrolysate; _diff_, difference; HR, Heart rate; bpm, beats pr. min; RER, respiratory exchange ratio

The correct versions of both Tables can be viewed ahead in this Correction article.
